# Inhibition of the *ITGB1* gene attenuates crystalline silica-induced pulmonary fibrosis via epithelial-mesenchymal transformation

**DOI:** 10.1590/1414-431X2024e13486

**Published:** 2024-09-06

**Authors:** Haibin Li, Shushuo Xu, Xinxiao Li, Penghao Wang, Meng Hu, Ning Li, Qiang Zhou, Meiyu Chang, Sanqiao Yao

**Affiliations:** 1 School of Public Health, North China University of Science and Technology, Tangshan, China; 2 School of Public Health, Xinxiang Medical University, Xinxiang, China

**Keywords:** Silicosis, EMT, ITGB1, Integrin/ILK signaling pathway, CRISPR/Cas9

## Abstract

Silicosis is a systemic disease caused by long-term exposure to high concentrations of free silica dust particles in the workplace. It is characterized by a persistent inflammatory response, fibroblast proliferation, and excessive collagen deposition, leading to pulmonary interstitial fibrosis. Epithelial interstitial transformation (EMT) can cause epithelial cells to lose their tight junctions, cell polarity, and epithelial properties, thereby enhancing the properties of interstitial cells, which can lead to the progression of fibrosis and the formation of scar tissue. Integrin 1 (ITGB1) is considered an important factor for promoting EMT and tumor invasion in a variety of tumors and also plays an important role in the progression of fibrotic diseases. Therefore, ITGB1 can be used as a potential target for the treatment of silicosis. In this study, we found that silica exposure induced epithelial-mesenchymal transformation in rats and that the expression of integrin ITGB1 was elevated along with the EMT. We used CRISPR/Cas9 technology to construct integrin ITGB1 knockdown cell lines for *in vitro* experiments. We compared the expression of the EMT key proteins E-cadherin and vimentin in the ITGB1 knockdown cells and wild-type cells simultaneously stimulated by silica and detected the aggregation point distribution of E-cadherin and vimentin in the cells using laser confocal microscopy. Our results showed that ITGB1 knockout inhibited the ITGB1/ILK/Snail signaling pathway and attenuated the EMT occurrence compared to control cells. These results suggested that ITGB1 is associated with silica-induced EMT and may be a potential target for the treatment of silicosis.

## Introduction

Silicosis is a disease marked by widespread nodular fibrosis of the lungs brought on by prolonged inhalation of a significant quantity of dust that contains free silica (1). In addition, with the development of modern industrialization, there are many opportunities for people to come into contact with silica when engaged in various types of productive labor. Dust is widespread in all industrial systems, such as mining, infrastructure, and road construction. People working in these environments with inadequate protection are at an increased risk of acute or sudden silicosis. Even without exposure to dust, silicosis can worsen lung damage and, more seriously, can lead to respiratory failure and even death (2). Despite advances in protective methods, silicosis remains a persistent problem (3). Therefore, it is necessary to study its occurrence, development, and molecular mechanisms of action.

Silicosis, characterized by long-term chronic alveolitis and pulmonary interstitial fibrosis, is characterized by a complex cell-cytokine interaction (4). Silicotic fibrosis is the process of epithelial interstitial transformation (EMT) during tissue repair (5). Both animal and *in vitro* experiments have shown that EMT occurs during the silicosis-fibrosis process (6). Many vimentin (laser confocal cell marker for myoblasts)- and FSP-1 (fibroblast marker)-positive cells were observed in silicon nodules, confirming the differentiation of bronchoalveolar epithelial cells into myoblasts during silicosis (7).

Integrin 1 (ITGB1) is a subfamily of integrins present in various eukaryotic cells. It is a transmembrane protein that acts as an integrin receptor and plays a crucial role in cell adhesion and migration (8). In contrast, ITGB1 and EMT are essential in cell biology; EMT consists of physiological mechanisms that transform epithelial cells into mesenchymal phenotypes, resulting in greater mobility and aggressiveness (9).

Theys et al. (10) found that integrin is closely related to the occurrence of EMT and that the upregulation of integrin expression can promote the development of EMT and fibrosis. Walker and Menko (11) found that integrin αvβ3 promoted the occurrence of EMT by regulating the TGF-β receptor and that TGF-β could not induce EMT when integrin β3 was deficient. Studies have shown that ITGB1 promotes the occurrence and progression of EMT by regulating various signaling pathways, such as PI3K/AKT, MAPK, and Wnt (12). ITGB1 affects the expression and activity of the transcription factors Snail, Slug, and Twist (13), which are major regulators of EMT. Moreover, ITGB1 can bind to the extracellular matrix and promote the remodeling and rearrangement of the extracellular matrix, thus affecting changes in cell morphology and function, which is an important event in the EMT process (14). ITGB1 is involved in the occurrence and progression of EMT in various ways and regulates the physiological processes of cell proliferation, migration, adhesion, and invasion. This is considered an important factor in promoting EMT and invasion in various tumors (15).

ITGB1 and EMT are closely related biological processes crucial for various pathological and physiological mechanisms. However, the function of ITGB1 in silicosis remains unclear. Therefore, in this study, we assessed the relationship between ITGB1 and EMT and the role of ITGB1 in silicosis fibrosis. We also present a theoretical foundation and a potentially effective molecular target for the treatment of fibrosis.

## Material and Methods

### Reagents and antibodies

The CRISPR/Cas9 skeleton vector pll3.7-mU6-CMV-NLShuCAS9-NLS (Cas9-wild) was amplified to RNA (guide RNA); the gRNA.opti and SSA-RPG reporter vectors were obtained from the laboratory of Professor Zhang Zhiying (College of Animal Science, China). The following products were also used to carry out the experiments: restriction endonuclease (New, England Biolabs, USA); Protein ladder (Thermo Fisher Scientific, 26617, USA); DNA marker (Beijing Quanshi Gold Biotechnology Co., Ltd., BM121, China); plasmid extraction kit (American Omega, D6943-01*, USA); agarose gel recovery kit (Omega, D250-01); ITGB1 antibody (Affinity Biosciences, USA); E-cadherin antibody (Affinity Biosciences); vimentin antibody (Affinity Biosciences); ILK antibody (Affinity Biosciences); Snail antibody (Affinity Biosciences); GAPDH antibody (Beijing Boosen Biotechnology Company, China); fetal bovine serum (Gibco, USA); and DMEM high glucose medium (Gibco).

### Animal models of pulmonary fibrosis

**Figure 1:**
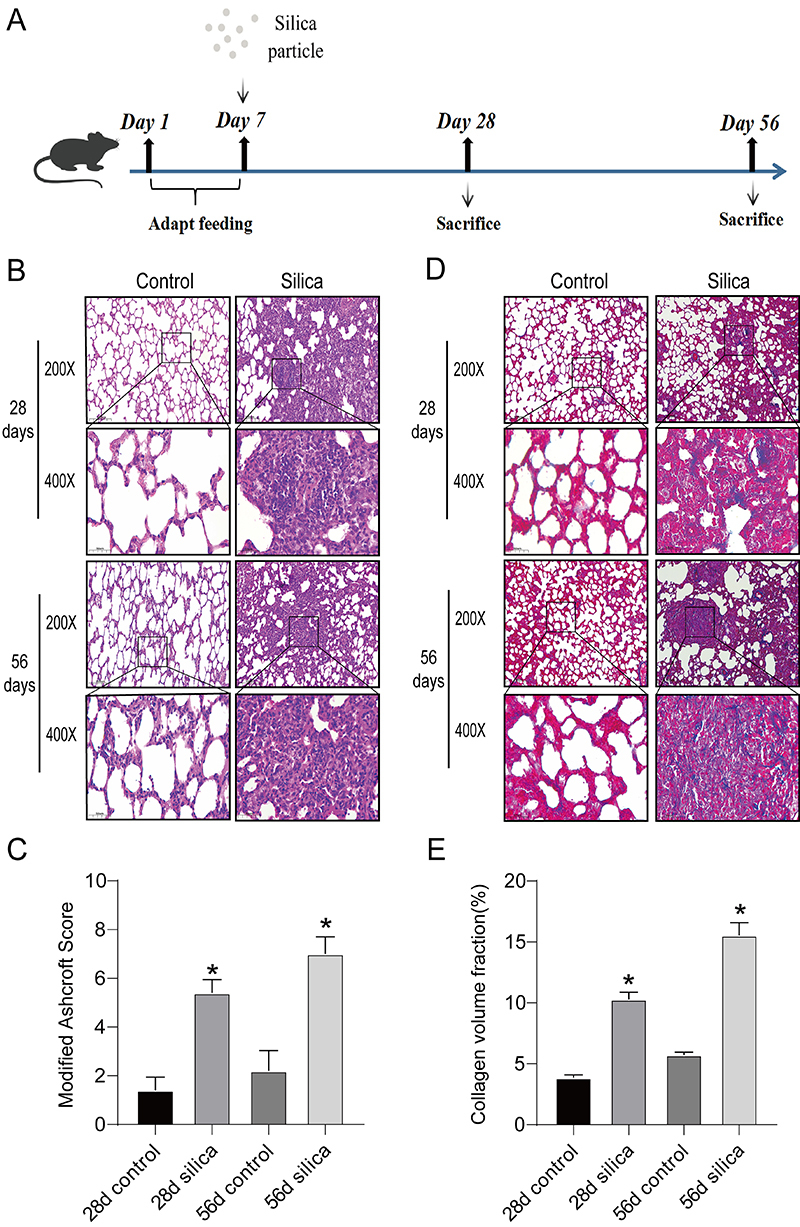
Effects of silica induction on lung pathology and collagen deposition in rats. **A**, Schematic diagram showing the experimental animal procedure. **B**, Hematoxylin-eosin staining of rat lung tissues at 28 and 56 days following silica induction. **C**, The modified Ashcroft score was used to estimate the pathology of pulmonary fibrosis. **D**, Masson staining of rat lung tissues at 28 and 56 days following silica induction. **E**, Collagen volume fraction. **C** and **E**, Data are reported as means±SD (n=3). *P<0.05 *vs* saline control group (ANOVA). Scale bars 50 and 100 μm.

Sipford Biotechnology Co., Ltd. (China) provided 40 mature male SPF SD rats (5-7 weeks old, 180-200 g). The Experimental Animal Production License No is SCXK 2019-0010. All the rats were kept in an SPF environment, and the feeding conditions complied with the GB 14925-2010 Environment and Facilities for Experimental Animals guideline. The animals were divided into two categories: silicosis models and standard saline controls. An intrapulmonary silica suspension (1.5 mL, 50 g/L; Xinxiang Medical University, China) was administered to the silicosis model group (16,17), and equal amounts of sterile saline were administered to the control group. The rats were divided into two groups of ten each on days 28 and 56 and lung tissues of the mice were collected on days 28 and 56 (Figure 1A).

### HE and Masson staining

The lung tissue was preserved in 4% paraformaldehyde, and paraffin slices were prepared 24 h later. The embedded tissue was sliced into 4-μm segments, and Masson as well as hematoxylin and eosin (HE) staining were carried out. Histopathological images were obtained using an upright light microscope (Leica DM3000, Germany). The modified Ashcroft histopathology score was used to determine the fibrosis score (18). Moreover, Masson's tricolor collagen deposition was measured using an Image-Pro Plus 6 (Media Cybernetics Inc., USA).

### Immunohistochemical staining

Rat lung tissues were sliced, dewaxed, sealed, decolorized, and rinsed. Subsequently, the membranes were blocked with 5% bovine serum albumin (BSA). Rabbit anti-ITGB1 (1:100), vimentin (1:500), and E-cadherin (1:500) were kept in an incubator overnight at 4°C with the paraffin slices and were stained with DAB and hematoxylin following treatment with goat anti-rabbit IgG-horseradish peroxidase (1:200) for 1 h. An upright optical microscope (Leica DM3000) was used to view the images, and brown particles were recorded as positive cells. The images were subjected to quantitative analysis using Image-Pro Plus 6.

### Cell culture and treatment

BEAS-2B, a human bronchial epithelial cell line, was obtained from the American Type Culture Collection and grown in Dulbecco's modified DMEM medium (DMEM) supplemented with 1% penicillin-streptomycin and 10% FBS (Gibco). Two groups of BEAS-2B cells and ITGB1 knockdown cells were cultured in culture dishes at 50,000 cells per dish. They were divided into control (containing BEAS-2B cells, ITGB1 knockdown cells, and 1000 UL DMEM base medium) and experimental groups (containing BEAS-2B cells, 1000 UL DMEM base medium, ITGB1 knock-down cells, and 100 μL SiO_2_) (16). After adhesion, cells were treated with a fresh basic medium for 24 h.

### Creation of the ITGB1^-/-^-BEAS-2B cell line

The first target T1 sequence AATGTAACCAACCGTAGCAAAGG and the second target T2 sequence AATGCCTCAAGTAAACACGCAGG were obtained from National Center for Biotechnology Information (https://www.benchling.com/academic/). gRNA1 and gRNA2 were inserted into the CRISPR/Cas9 expression vector to obtain ITGB1-1 CRISPR/Cas9 and ITGB1-2 CRISPR/Cas9, respectively, targeting the *ITGB1* gene. The CRISPR/Cas9 expression vector was pX330-U6-Chimeric_BB-CBh-hSpCas9 (Addgene, USA). The ligand product was transformed into the receptor bacteria DH5α using the enzyme digestion method, and the plasmid was extracted according to the instructions of the plasmid extraction kit (American Omega). The Lipofectam 2000 transfected cells were cultured in a CO_2_ incubator at 37°C. For seven days, 0.5 µg/mL puromycin was added to the medium. After the cells were digested with trypsin, they were cultured in a 100-cm petri dish at a density of 10 cells/dish. Once the cells grew into monoclones, 50 monoclones were selected for an expanded culture, and anti-purinomycin monoclones were selected for DNA extraction and PCR amplification. Positive cells were screened by agarose electrophoresis, namely the *ITGB1* gene knockdown BEAS-2B cell line.

### Mutation detection

Mutant single-cell clones and wild-type BEAS-2B cells were collected, washed twice using PBS, and extracted by sodium dodecyl sulfate. Genomic DNA was obtained from each group and amplified using PCR. The primer information is: F: GGTAAATGCGAGAATGATCCT, R: GTCAAGAAGGCACCATAGCTG. The PCR reaction procedure was pre-denaturation at 95°C for 5 min, denaturation at 95°C for 30 s, annealing at 60°C for 30 s, extension at 72°C for 50 s, 25 cycles, and finally extension at 72°C for 10 min. Next, 2% agarose electrophoresis (110 V for 40 min) was performed, and the ECL chemiluminescence method was used to develop the image.

### Western blotting analysis

Following cell culture, the ice lysate protein was used in a lytic solution (RIPA+PMSF). Centrifugation was performed at 13,400 g for 15 min at xx°C and the total protein was extracted. The protein content was determined using the Bradford method, and the protein samples were homogenized by SDS-PAGE (90 V, 30 min; 120 V, 50 min). Proteins were transferred to a PVDF membrane with a pore size of 0.45 um (300 mA, 70 min) for 2 h with 5% skim milk and thereafter treated with the primary antibody ITGB1 (1:1000) (Proteintech, USA). E-cadherin (1:5000) (Affinity Biosciences), vimentin (1:5000) (Affinity Biosciences), ILK (1:1000) (Proteintech), Snail (1:1000) (Affinity Biosciences), and GAPDH (1:1000) (Affinity Biosciences) were added, and the samples were incubated for 2 h. Next, Tween (TBST) buffered with 5% Tris was used to rinse the samples three times, lasting ten minutes each. They were then incubated with HRP-conjugated secondary antibodies (1:5000) (Affinity Biosciences) for 1 h and thereafter rinsed three times with TBST once again. Finally, the images were developed using the ECL chemiluminescence method, exposed to a gel imaging system, and analyzed using the ImageJ software (NIH, USA).

### Cell proliferation assays

The wild-type and ITGB1^-/-^-BEAS-2B cells were counted at 5×10^4^ cells/well at 12, 24, 36, and 48 h. Cells were counted in the field of view and the well. Each experiment was repeated three times in parallel. Cells from the different groups (2×10^3^ cells/well) were grown in DMEM supplemented with 10% fetal bovine serum after being injected into 96-well culture plates. After 12, 24, and 36 h of culture, 10 μL of cell counting kit-8 (CCK-8) solution (Dojindo, Japan) was added to every well and the plates were incubated for an additional 2 h at 37°C. An EnSpire Multimode Plate Reader (Thermo Scientific Multiskan GO, USA) was used to detect spectral absorbance at 450 nm.

### Immunofluorescence

Liver cells were seeded onto 12-well plates and cultured for 4-6 h before drug intervention. After the intervention, the waste solution was removed, and the cells were rinsed three times with PBS before being fixed with 4% paraformaldehyde for 30 min; following this, they were washed three times once again. Subsequently, 3% Triton X-100 was incubated with cells at room temperature (25°C) for 30 min, after which they were cleaned again. After blocking with 1% BSA for 30 min, the product was sucked out, and the primary antibodies E-cadherin (1:200) and vimentin (1:200) were added to it overnight at 4°C for 16 h. After three washes at room temperature (25°C) for 1 h, goat anti-rabbit IgG labeled with horseradish peroxidase was applied, and the cells were stained with DAPI and viewed under a confocal laser microscope (Leica SP8 STED3X).

### Cell migration assay

BEAS-2B cells were cultivated. When cell development reached 90%, a 200-mL sterile spearhead was used to make a scratch in the middle of the plate. The loose cells were then washed with PBS and the plate incubated for 24 h. Cell migration was observed and photographed under a microscope (CX23, Olympus, Japan) at various time points. Relative cell mobility was calculated by dividing the scratch area after 24 h of treatment by the scratch area before treatment.

### Statistical analysis

SPSS version 25.0 (IBM, USA) was used to analyze all the experimental data. The data are reported as means±SD, and the differences between multiple groups were compared using one-way analysis of variance, followed by Tukey's multiple comparisons test. Statistical significance was set at P<0.05.

## Results

### SiO_2_-induced lung inflammation and fibrosis in rats

HE and Masson staining were performed on the rat lung tissue sections (Figure 1B). HE staining revealed that after 28 days, no inflammatory cells were observed within the normal saline group (control rats), and the lung tissue architecture was typical. In contrast, the alveolar structure was distorted, and inflammatory cells and fibrotic nodules were observed in the rat lung tissue after 28 days. After 58 days, lung tissue structure of rats in the regular saline control group was normal with mild alveolar interstitial inflammation. In the lung tissue of the silicosis model group, prominent silicone nodules were observed, together with a significant number of inflammatory cells and a thickened cell wall (Figure 1C).

Masson's trichrome staining (Figure 1D) revealed that the interstitium of the regular saline control group showed no discernible dispersion of blue collagen. Conversely, in the silicosis experimental group, the lung interstitium showed a significant amount of blue collagen deposition after 28 days. After 56 days of treatment with the standard saline control, no blue collagen fibers were produced in the rat lung tissues. However, the lung structure of the 56-day silicosis model group was seriously damaged and the number of blue collagen fibers significantly increased (Figure 1E). These findings suggested that silica caused collagen deposition, fibrosis, and inflammation in rats.

### EMT and ITGB1 proteins were abnormally expressed following silica exposure

**Figure 2:**
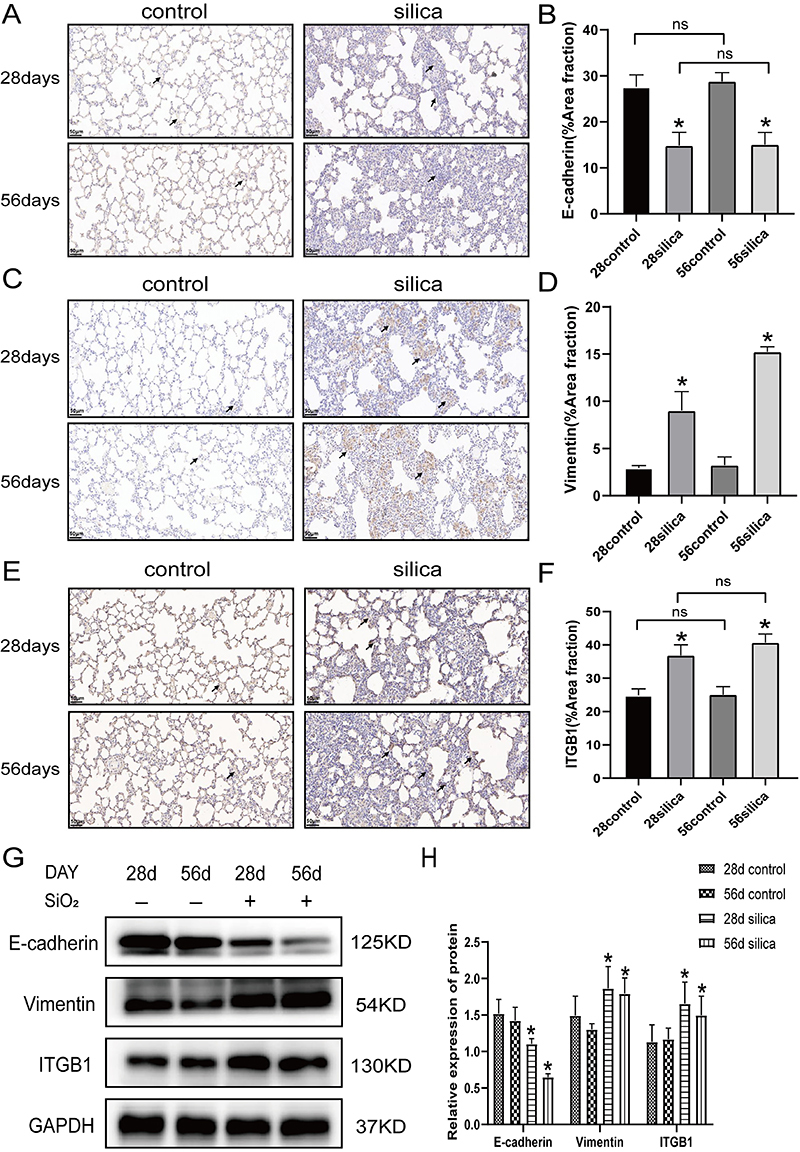
Immunohistochemical staining and western blot following epithelial interstitial transformation (EMT) induced by silica in rats. **A**, **C**, and **E**, Representative images of E-cadherin, vimentin, and ITGB1 positive expression in lung tissue (scale bars 50 μm). The arrows indicate E-cadherin-, vimentin-, and ITGB1-positive cells in IHC. **B**, **D**, and **F**, Statistical analysis of **A**, **C**, and **E**. **G**, Western blotting was used to detect the expression of EMT markers and ITGB1 in rat lung tissues of the control and experimental groups. **H**, Protein levels of E-cadherin, vimentin, and ITGB1. Data are reported as means±SD (n=3). *P<0.05 *vs* saline control group; ns: not significant (ANOVA).

By analyzing lung slices using immunohistochemistry, the EMT induced by silica in the rat lung tissue was further explained. The alveolar walls in the saline control group were surrounded by numerous E-cadherin-positive cells (Figure 2A and B). In contrast, the expression of vimentin was limited to the smooth muscles and blood vessels of the trachea. (Figure 2C and D). In the region of severe fibrosis, namely, silicon nodules, the vimentin-positive cells in the silicosis model group were more common than that in the ordinary saline control group, and the positive expression of E-cadherin decreased significantly. These findings suggested that silica induces EMT in rats. An immunohistochemical examination of ITGB1 protein was performed to examine the expression of ITGB1 in the SiO_2_-induced rat lung tissue (Figure 2E and F). The silicosis rat model group expressed ITGB1 at much higher levels than the saline control group. These results showed that silica induction boosted ITGB1 expression in the rat alveoli. Next, we extracted the rat lung tissue for western blotting, and the outcomes matched those of the immunohistochemistry (Figure 2G and H).

### *ITGB1* gene knockdown BEAS-2B cell line

**Figure 3:**
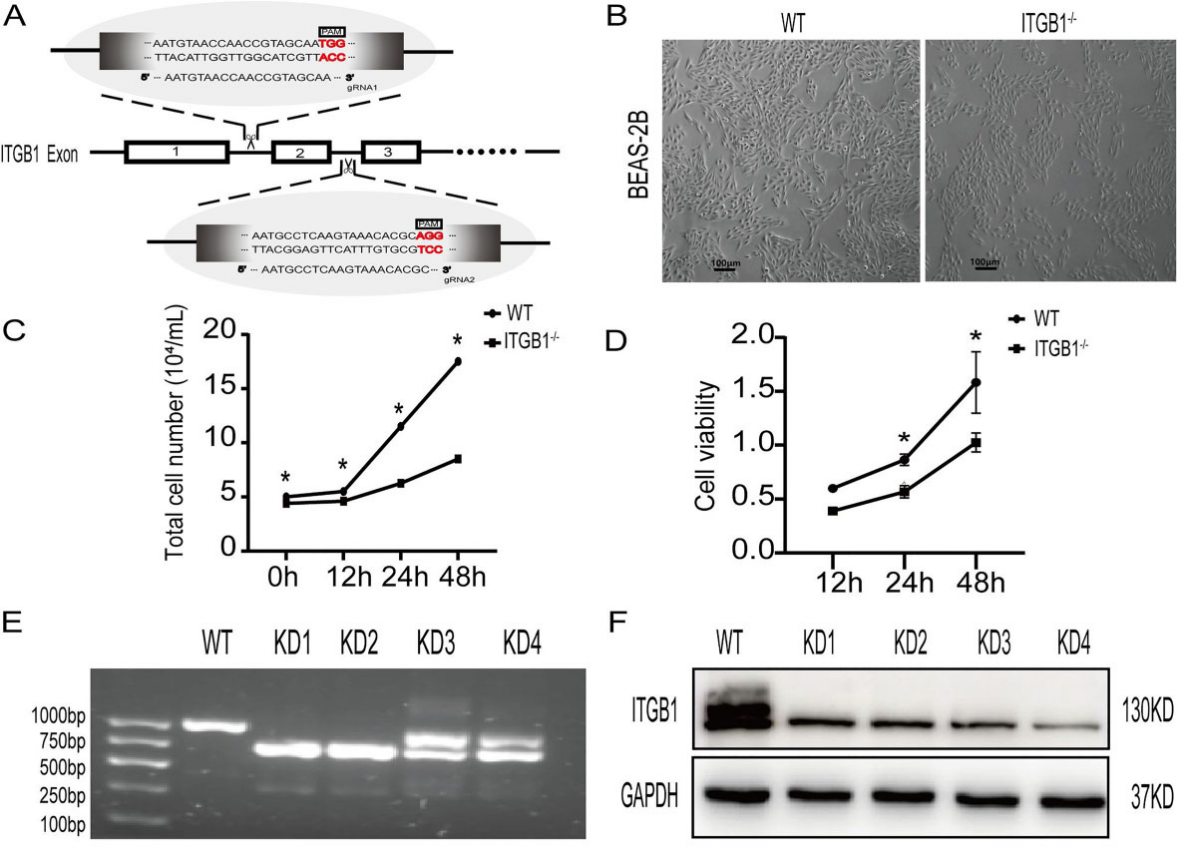
Validation of ITGB1 knockdown (KD) in BEAS-2B cells. **A**, sgRNA targets are located before and after the second exon of the *ITGB1* gene. The figure shows the sequences containing the gRNA primers T1 and T2. **B**, After 24 h, the control (WT) and experimental groups were examined under a microscope. BEAS-2B cells appeared cubic and polygon-shaped, typical of respiratory epithelial cells. When ITGB1 was knocked down, BEAS-2B cells were short and fusiform. Scale bars 100 μm. **C**, The vitality of cells was determined using a counting cell test in the presence and absence of the *ITGB1* gene (n=3). **D**, Cell proliferation assay was performed using the Cell Counting Kit-8 according to the manufacturer's instruction. **E**, Agarose gel electrophoresis confirmed the KD of ITGB1. **F**, Western blot assay was used to detect ITGB1 protein expression in the control and experimental groups. Data are reported as means±SD. *P<0.05 (ANOVA).

We established ITGB1 knockdown cells in BEAS-2B cells using CRISPR/Cas9 technology. Based on human gene sequences from the NCBI database, we determined gRNA primers targeting ITGB1: first target sequence T1 and second target sequence T2 (Figure 3A). gRNA1 and gRNA2 were inserted into the CRISPR/Cas9 expression vector to generate ITGB1-1 CRISPR/Cas9 and ITGB1-2 CRISPR/Cas9 aimed at ITGB1. The cells were simultaneously transfected into BEAS-2B cells and subjected to purinomycin screening. We observed morphological changes in the monoclonal cells compared to the wild-type cells under a microscope (Leica DM3000) (Figure 3B). The total number of the two cell types at 0, 12, 24, and 48 h was recorded by cell counting, and cell growth curves were drawn. We observed that total cell proliferation in the wild-type cells after 24 and 48 h was much greater than that in the monoclonal cells (Figure 3C). In addition, a CCK-8 assay was used to analyze the growth rates of the two cell types. After 48 h of monitoring, the monoclonal cell proliferation was lower than that of the wild-type cells (Figure 3D). We then selected four monoclones from which to extract DNA for PCR amplification and agarose electrophoresis. ITGB1 was knocked down in the monoclonal cells (Figure 3E). Western blot analysis showed that the ITGB1 protein expression was very low in the mutant monoclonal cells (Figure 3F).

### Gene knockdown cells inhibited SiO_2_-induced EMT via the integrin/ILK/Snail pathway

**Figure 4:**
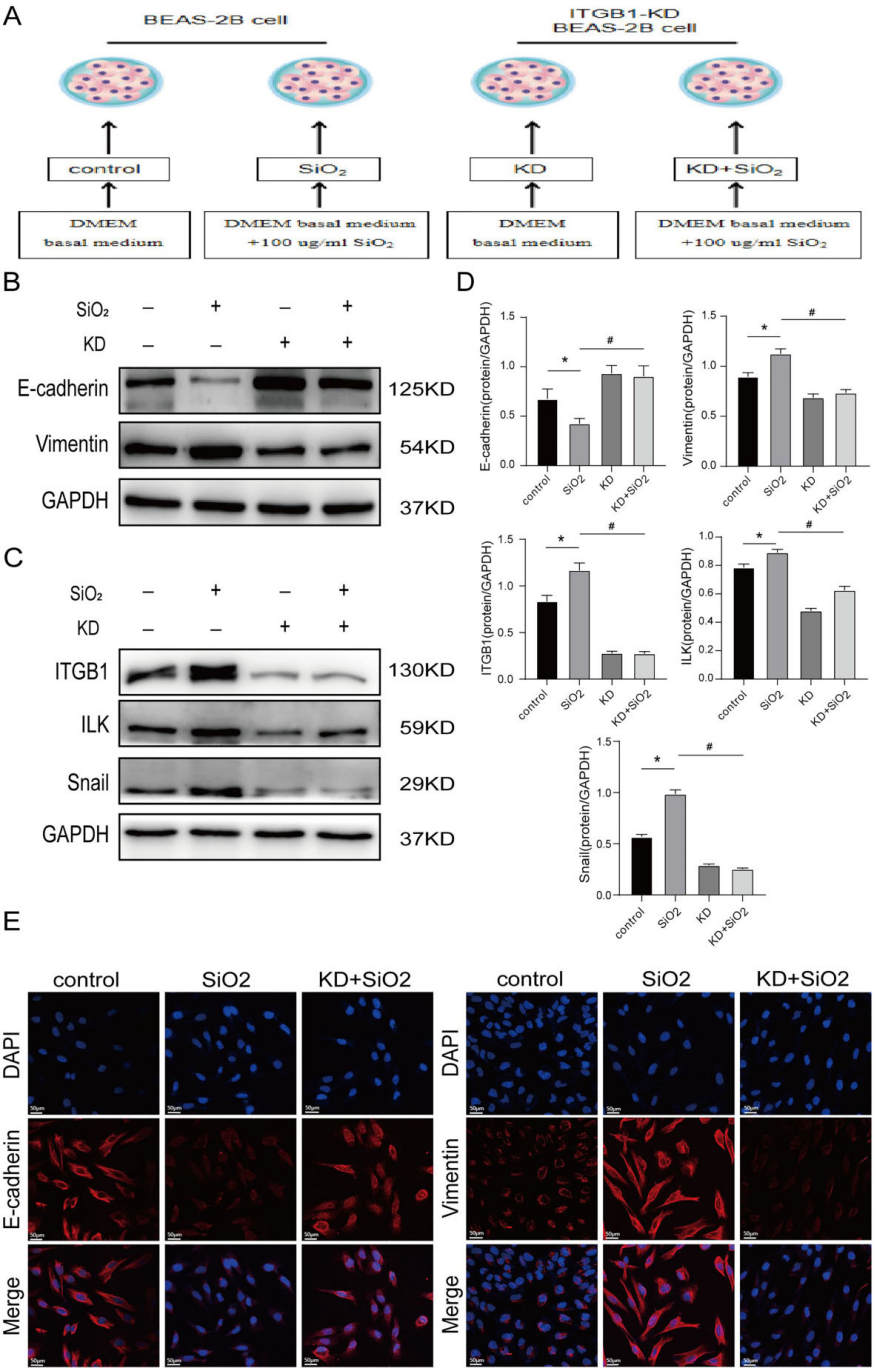
Effect of knockdown (KD) ITGB1 on integrin/ILK signaling pathway and epithelial interstitial transformation (EMT) in silica-stimulated BEAS-2B cells. **A**, Schematic diagram of the experimental cells. **B**, Western blotting detected the expression of EMT markers in the ITGB1-knocked down BEAS-2B cells. **C**, The expression of integrin/ILK signaling pathway markers in BEAS-2B cells treated with ITGB1^-/-^ was measured by western blotting. **D**, The protein levels of E-cadherin, vimentin, ITGB1, ILK, and Snail were quantified by the ImageJ 6.0 software. Data are reported as means±SD (n=3). *P<0.05 *vs* control group, ^#^P<0.05 *vs* SiO_2_ group (ANOVA). **E**, Confocal microscopy was used to observe the immunofluorescence of E-cadherin and vimentin. Scale bars 50 μm.

The experimental group was further divided into four groups: control, SiO_2_ model, knock-down (KD), and KD+SiO_2_ (Figure 4A). Western blotting showed that in contrast to the control group (Figure 4B and D), the expression of the E-cadherin protein in the SiO_2_ model group decreased, vimentin protein expression increased, EMT occurred, and the integrin/ILK/Snail pathway protein expression increased (Figure 4C and D). Compared to the KD+SiO_2_ group, E-cadherin expression increased, vimentin expression decreased, and the integrin/ILK/Snail pathway protein expression decreased. We also performed protein immunofluorescence experiments and confocal microscopy, which revealed similar results (Figure 4E). The positive expression of E-cadherin in the KD+SiO_2_ group was more significant than that in the SiO_2_ model group. In comparison, vimentin expression was significantly lower. These findings suggested that the knockdown of ITGB1 not only reduced the expression of the integrin/ILK/Snail pathway but also impeded the SiO_2_-induced EMT of the BEAS-2B cells.

### BEAS-2B cell migration was suppressed by gene knockdown

BEAS-2B cells did not migrate to a typical physiological environment. However, cells changed from epithelial to mesenchymal when stimulated by silica crystals, thereby gaining the capacity to move. We investigated how ITGB1 knockdown and silica intervention affected BEAS-2B cell migration by a scratch assay. Three groups were involved in the experiment: the KD+SiO_2_, SiO_2_, and control groups. The SiO_2_ model group's relative cell mobility rose significantly compared to that of the control group, and the ability of the group to migrate to the scratch areas was improved.

**Figure 5:**
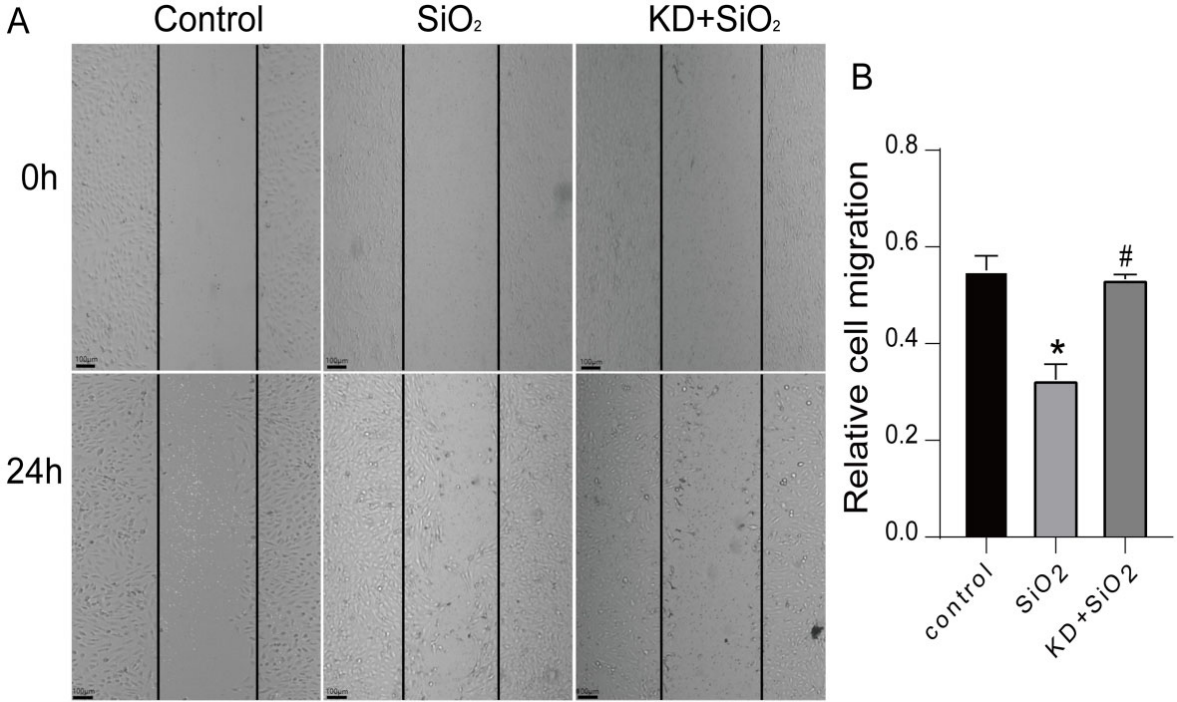
The effect of knockdown (KD) integrin ITGB1 on the migration of BEAS-2B cells. **A**, BEAS-2B cells were migrated at 0 h and 24 h in each group (scale bars 100 μm). **B**, The migration of BEAS-2B cells in each group was quantified by the ImageJ 6.0 software. Data are reported as means±SD (n=3). *P<0.05 *vs* control group, ^#^P<0.05 *vs* SiO_2_ group (ANOVA).

However, the KD+SiO_2_ group's migration to the scratch zone decreased compared to that of the SiO_2_ model group, and the relative mobility of the cells decreased dramatically (Figure 5A and B). Based on these findings, the BEAS-2B cell migratory capacity may be enhanced by silica stimulation and decreased by ITGB1 knockdown.

## Discussion

In this study, we found that silica exposure induced epithelial interstitial transformation and fibrosis formation *in vivo* in rat lung tissue and that EMT is an important step of silicosis-induced fibrosis pathogenesis. ITGB1 knockdown in BEAS-2B cells inhibited silica-induced EMT *in vitro*. Mechanistically, the downregulation of integrin ITGB1 may be a viable strategy for treating silica-induced pulmonary fibrosis. This study can also serve as a theoretical basis for the development of new treatments that will contribute to the definition of pulmonary fibrosis and future clinical treatment options.

Silicosis has drawn increasing attention in China due to the high prevalence of pulmonary fibrosis caused by repetitive exposure to excessive silica dust and the lack of effective preventative and therapeutic measures (19). It is exciting that more and more research demonstrates that EMT is critical for several disorders related to fibrosis and that inhibiting EMT can reduce the damage caused by fibrosis, but the problem is that the regulatory molecular mechanism of EMT has not been fully elucidated (20,21). Previous studies have shown that the integrin/ILK pathway is closely associated with EMT (22). Based on previous studies, we hypothesized that the integrin/ILK signaling pathway might be specifically inhibited to mitigate the effects of silica-induced fibrosis and EMT. Therefore, in this study, we investigated the regulatory mechanism of ITGB1 knockdown therapy on EMT and the effect of silica on fibrosis via the integrin/ILK pathway.

Numerous *in vivo* and *in vitro* studies have demonstrated the role of EMT in silicosis fibrosis (23). In this study, we observed a large number of double-positive vimentin and FSP-1 cells in the silicone nodules using confocal laser microscopy (24,25). Furthermore, our findings revealed that bronchoalveolar epithelial cells and myofibroblasts differentiated during silicosis. According to prior research (16), E-cadherin protein expression is reduced and vimentin protein is increased in rat lung tissue exposed to SiO_2_ using immunohistochemistry and immune protein observation. This suggests that after silica induction, rats experience EMT.

The expression of ITGB1, the most highly expressed integrin, was significantly reduced in HCT116 and SW620 cells by ropivacaine, which suppressed colorectal cancer cell proliferation, migration, and invasion (26). At present, the available data show that Linc is highly effective in breast cancer and gallbladder cancer, and ITGB1-mediated cell migration and invasion are involved in the epithelial-mesenchymal transformation process (26,27). However, the details of the regulation of EMT by linc-ITGB1 have not been clarified, and we successfully managed to knockdown the *ITGB1* gene in BEAS-2B cells using CRISPR/Cas9 technology and evaluated the proliferation rate of ITGB1^-/-^-BEAS-2B cells using cell growth curves and the CCK-8 method, intuitively confirming that the knockdown of ITGB1 harms the proliferation of BEAS-2B cells.

The role of the ITG family in fibrosis is widely recognized, and data supporting the role of ITGB1 are increasing. By suppressing the expression of ITGB1, CSN5 deletion slows the proliferation of liver cancer cells (28). When an appropriate unit or ligand interacts with ITGB1, it triggers important molecules that give cancer cells the ability to adhere and metastasize (29), and to undergo EMT. According to our research, ITGB1 may interact with silica-induced EMT in rats, a finding supported by western blotting and immunohistochemistry. ITGB1 knockdown significantly delayed the proliferation and migration of BEAS-2B cells. In addition, the most well-known example is the activation of the integrin/ILK signaling pathway, which is linked to the regulation of EMT in the pathogenesis of fibrotic disorders and has attracted the interest of many researchers (30). According to other studies (31,32), ITGB1 can upregulate ILK expression, thereby activating the FAK and ILK signaling axes. Significantly elevated levels of phosphorylated FAK and ILK have been observed in MCF-10A cells overexpressing Twist and ITGB1 (33). Phosphorylated FAK and ILK levels decrease in Hs578T and BT549 cells upon the loss of Twist or ITGB1. The WNT, PI3K/AKT, MAPK/ERK, and other signaling pathways can all be inhibited by blocking the ITGB1-FAK/ILK signaling axis (34). Furthermore, it inhibits EMT and the metastasis of twist-positive breast cancer cells (35).

Our findings suggested that ILK is essential for integrin-mediated EMT. We found that ITGB1 activated the integrin/ILK signaling pathway. In the integrin signaling pathway, ILK acts as a bridge between integrin and the downstream signaling molecules (36). Furthermore, it is the meeting point of multiple intracellular signaling pathways through integrin β1 and β3 subunit binding and activation (37). Recently, it was found that fully activated ILK in tumor cells can inhibit Snail degradation, resulting in increased Snail content in cells. Snail is a zinc-containing DNA-binding protein that can be activated by E-box binding of the E-cadherin transcription factor activation site, blocking its transcription and downregulating E-cadherin expression (38). Increased Snail expression increases cell mobility and aggression by suppressing epithelial markers and increasing mesenchymal markers.

Previous studies have shown that the constitutive activation of ILK can lead to a carcinogenic phenotype in epithelial cells by downregulating E-cadherin expression and subsequent epithelial-to-mesenchymal transformation (39). Our study showed that SiO_2_ intervention increased the expression of ILK-activated ITGB1 and Snail proteins in BEAS-2B lung epithelial cells. The activation of the integrin/ILK/Snail signal is comparable to the up- and down-regulation of fibronectin vimentin expression and E-cadherin, suggesting that SiO_2_ intervention can lead to the EMT of lung epithelial cells by activating the integrin/ILK/Snail signaling pathway, which is confirmed in the BEAS-2B cells. In this study, we used *in vitro* silica stimulation of BEAS-2B cells as a positive control. We found that silica stimulated EMT in the BEAS-2B cells and activated the integrin/ILK pathway by increasing the Snail protein content. Following this, the ITGB1^-/-^-BEAS-2B cells were also prompted by silica western blot, and immunofluorescence experiments showed that ITGB1 knockdown treatment down-regulated the integrin/ILK pathway, reduced Snail expression, and suppressed EMT in the BEAS-2B cells.

**Figure 6:**
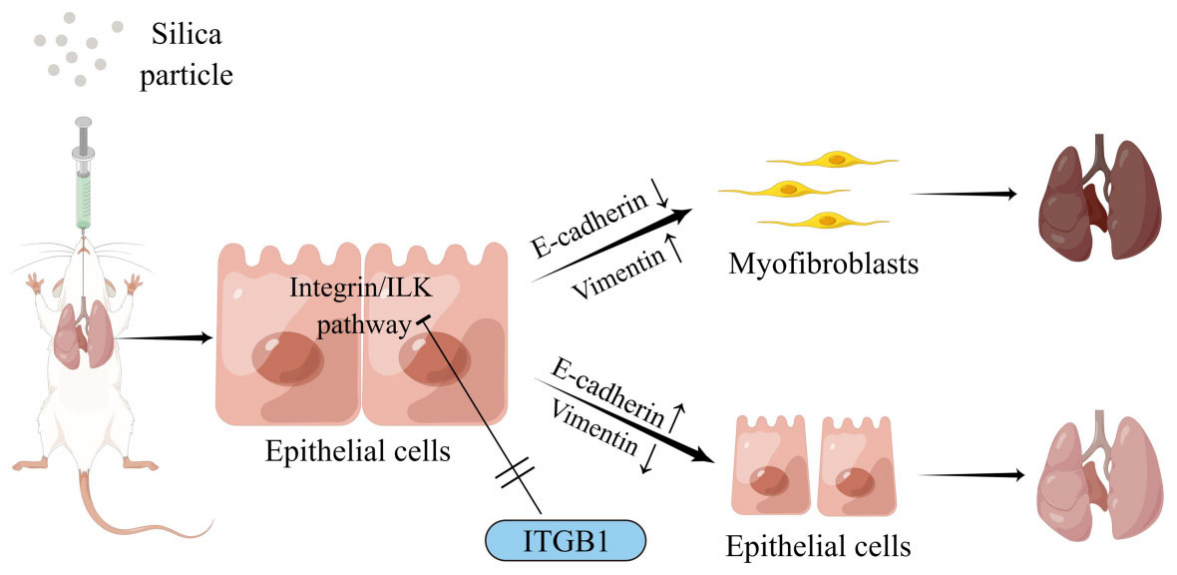
Role of the integrin/ILK signaling pathway in animal and cell models of epithelial interstitial transformation (EMT). *In vivo*, silica induced EMT in rats. *In vitro*, silica stimulated BEAS-2B cells to promote EMT by upregulating the integrin/ILK signaling pathway. ITGB1 knockdown reduced EMT by downregulating the integrin/ILK pathway.

In conclusion, we investigated the association between the EMT and ITGB1 expression. Through the integrin/ILK signaling pathway, silica can increase the expression of EMT-related proteins and promote the onset and progression of silicosis. CRISPR/Cas9 technology to knockdown ITGB1 can inhibit the integrin/ILK signaling pathway and alleviate silicosis fibrosis, offering a fresh perspective on silicosis therapy (Figure 6). However, it remains to be determined whether ITGB1 also interacts with other signaling pathways to influence the onset and progression of silicosis.
